# Earthworm extracts utilized in the green synthesis of gold nanoparticles capable of reinforcing the anticoagulant activities of heparin

**DOI:** 10.1186/1556-276X-8-542

**Published:** 2013-12-26

**Authors:** Hee Kyeong Kim, Myung-Jin Choi, Song-Hyun Cha, Yean Kyoung Koo, Sang Hui Jun, Seonho Cho, Youmie Park

**Affiliations:** 1 College of Pharmacy, Inje University, 607 Obang-dong, Gimhae, Gyeongnam 621-749, Republic of Korea; 2 National Creative Research Initiatives (NCRI) Center for Isogeometric Optimal Design, Seoul National University, 1 Gwanak-ro, Gwanak-gu, Seoul 151-744, Republic of Korea; 3 College of Pharmacy, Seoul National University, 1 Gwanak-ro, Gwanak-gu, Seoul 151-742, Republic of Korea

**Keywords:** Green synthesis, Earthworm extracts, Invertebrate natural products, Gold nanoparticles, Anticoagulant activity

## Abstract

Gold nanoparticles were obtained using a green synthesis approach with aqueous earthworm extracts without any additional reducing or capping agents. The gold nanoparticles were characterized using UV-visible spectrophotometry, high-resolution transmission electron microscopy, atomic force microscopy, field emission scanning electron microscopy, X-ray diffraction, Fourier transform infrared spectroscopy, and inductively coupled plasma mass spectrometry. The anticoagulant activity of the gold nanoparticles was assessed using the activated partial thromboplastin time and was mildly enhanced by combining the gold nanoparticles with heparin. In addition to the generation of spherical nanoparticles with an average diameter of 6.13 ± 2.13 nm, cubic and block-shaped nanoparticles with an average aspect ratio, defined as the length divided by width, of 1.47 were also observed.

## Background

Recent advances in nanotechnology have enabled the exploration of nanomaterials for diverse applications. Among the variety of nanomaterials, gold nanoparticles (AuNPs) are of considerable interest due to their versatility and potential uses in chemistry, biology, medicine, and pharmaceuticals. AuNPs possess numerous advantages, such as low cytotoxicity, facile modification of their surfaces, straightforward synthetic processes, and excellent biocompatibility [1,2]. Currently, research interest in gold nanomedicine is rapidly expanding. In 2010, approximately 14% of all publications on nanomedicine were directly related to gold nanomedicine [3].

The common approach for synthesizing AuNPs employs sodium citrate and/or sodium borohydride as reducing agents to convert gold salts into AuNPs. The emergence of sustainability initiatives has increased the use of biological entities as reducing agents in AuNP synthesis (i.e., green synthesis) to replace toxic chemicals. Many authors have extensively reported the green synthesis of AuNPs using diverse biological entities. These green synthetic processes are rapid, eco-friendly, and cost-effective, and they can easily be scaled up [4-8]. Examples of these diverse biological entities include plant extracts, polysaccharides, bacteria, fungi, yeasts, DNA, RNA, proteins, and polypeptides. We used aqueous earthworm (*Eisenia andrei*) extracts as a reducing agent for the green synthesis of AuNPs (EW-AuNPs). Earthworm extracts reportedly have anticoagulant, fibrinolytic, and antithrombotic activities [9-14]. Trisina and co-workers reported that the protein extracts from *Lumbricus rubellus* are responsible for antithrombotic and thrombolytic activities [14]. In addition to proteins, glycosaminoglycans (chondroitin/dermatan sulfates and heparan sulfate) are also present in earthworm (*E. andrei*) extracts [15].

EW-AuNPs were characterized using UV-visible spectrophotometry, high-resolution transmission electron microscopy (HR-TEM), atomic force microscopy (AFM), field emission scanning electron microscopy (FE-SEM), X-ray diffraction (XRD), Fourier transform infrared spectroscopy (FT-IR), and inductively coupled plasma mass spectrometry (ICP-MS). As previously mentioned, anticoagulant activity is reportedly among the major biological activities of earthworm extracts; therefore, we assessed the anticoagulant activities of EW-AuNPs both alone and in combination with heparin.

## Methods

Hydrochloroauric acid trihydrate (HAuCl_4_ · 3H_2_O) and Minisart® syringe filters (0.45 μm; Sartorius AG, Goettingen, Germany) were obtained from Sigma-Aldrich (St. Louis, MO, USA). Earthworm (*E. andrei*) powders were obtained from a local supplier (Hwasun, Cheollanam-Do, Republic of Korea). Heparin sodium injection was obtained from JW Pharmaceutical (Seoul, Republic of Korea). All other reagents were of analytical grade. We previously reported the green synthesis of AuNPs using aqueous earthworm (*E. andrei*) extracts, the reaction process was optimized, and HR-TEM images of the AuNPs were obtained [16]. This procedure, with a minor modification, was utilized in this study. The earthworm powder (150 mg) was dispersed in deionized water (50 mL) and sonicated for 30 min. The insoluble pellet was removed after centrifugation at 5,067 × *g* for 10 min (Eppendorf 5424R centrifuge, Eppendorf AG, Hamburg, Germany). The supernatant was subsequently filtered through filter paper and a Minisart® filter (0.45 μm) and then freeze-dried. The freeze-dried material was used to synthesize the EW-AuNPs according to the following procedures: the earthworm extract (500 μL, 0.3% in deionized water) was mixed with HAuCl_4_ · 3H_2_O (500 μL, 0.6 mM in deionized water), and the mixture was incubated in an 80°C oven for 11 h. The reaction yield was measured by detecting the concentration of unreacted Au^3+^ via ICP-MS, which was conducted using an ELAN 6100 instrument (PerkinElmer SCIEX, Waltham, MA, USA). The samples containing unreacted Au^3+^ were prepared either by ultracentrifugation or by filtration. Ultracentrifugation was performed in an Eppendorf 5424R centrifuge at 21,130 × *g* for 1 h at 18°C. Under this ultracentrifugation condition, AuNPs remained as a wine-red pellet, and the color of the supernatant turned colorless. The supernatant containing the unreacted Au^3+^ was then pooled and analyzed via ICP-MS. The EW-AuNP solution was filtered through a syringe equipped with a Minisart® filter (0.45 μm). The colorless filtrate was also analyzed via ICP-MS. ICP-MS analysis was performed in triplicate to obtain an average yield. A Shimadzu UV-1800 spectrophotometer was used to acquire the UV-visible spectra (Shimadzu Corporation, Kyoto, Japan). A JEOL JEM-3010 TEM (JEOL Ltd., Tokyo, Japan) operating at 300 kV with samples on a carbon-coated copper grid (carbon type-B, 300 mesh, Ted Pella Inc., Redding, CA, USA) was used to obtain the HR-TEM. The AFM images were acquired using a Dimension® Icon® (Bruker Nano, Inc., Santa Barbara, CA, USA) with an RTESP probe (MPP-11100-10, premium high-resolution tapping-mode silicon probe, Bruker Nano, Inc., Santa Barbara, CA, USA) in tapping mode. The mica (grade V-1, 25 mm × 25 mm, 0.15-mm thick) was acquired from the SPI Supplies Division of Structure Probe, Inc. (West Chester, PA, USA) and was used for the sample deposition. FE-SEM images were obtained using a JSM-7100 F with an accelerating voltage of 15 kV (JEOL Ltd., Tokyo, Japan). The samples were lyophilized with a FD5505 freeze drier (Il Shin Bio, Seoul, Republic of Korea). The FT-IR spectra were acquired with a KBr pellet of the freeze-dried samples using a Nicolet 6700 spectrometer (Thermo Fisher Scientific, Waltham, MA, USA) over a range of 400 ~ 4,000 cm^−1^. XRD analysis was performed using a Bruker D8 Discover high-resolution X-ray diffractometer (Bruker, Karlsruhe, Germany) with CuKα radiation (*λ* = 0.154056 nm) over the range of 20° ~ 90° (2*θ* scale). A tenfold AuNP concentrate was processed under an N_2_ atmosphere to assess the activated partial thromboplastin time (aPTT) using a procedure adapted from our previous report [17].

## Results and discussion

### Green synthesis and yield of EW-AuNPs

**Figure 1:**
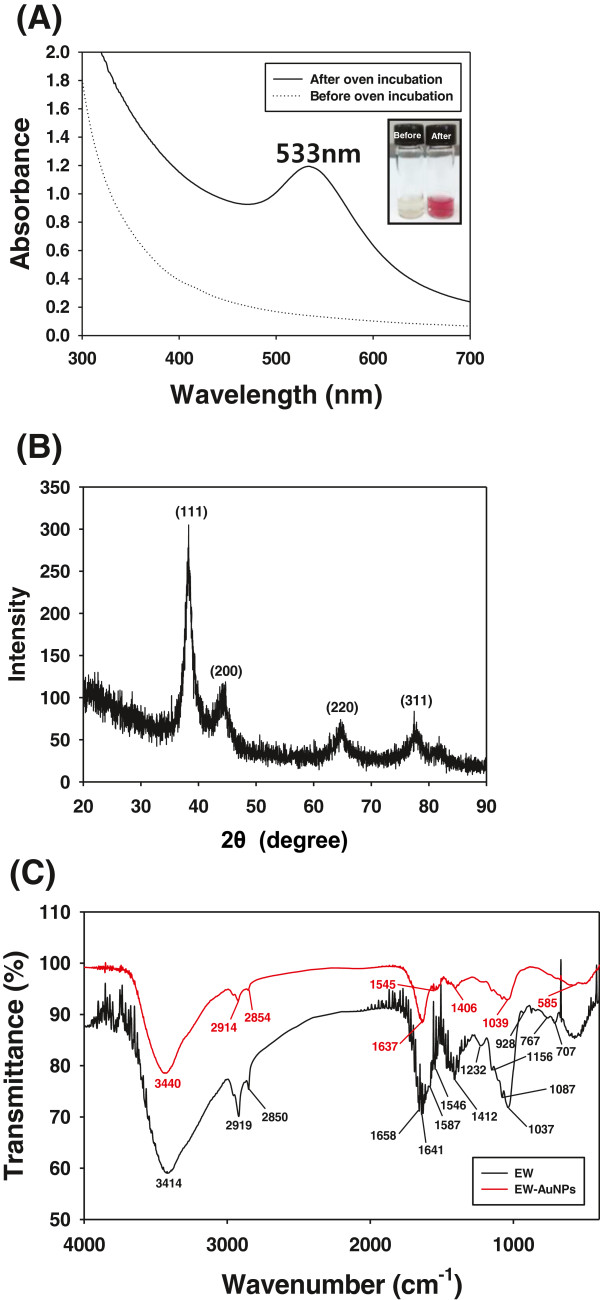
**UV-visible spectra, XRD analysis, and FT-IR spectra of EW-AuNPs. (A)** UV-visible spectra before and after the oven incubation. The inset depicts the color change of the AuNP solution. **(B)** XRD analysis of the EW-AuNPs. **(C)** FT-IR spectra of the EW and EW-AuNPs.

As depicted in Figure 1A, the wine-red color of the EW-AuNPs after incubation in an oven confirmed the successful synthesis of the AuNPs. The surface plasmon resonance band of AuNPs was observed at 533 nm. ICP-MS is an excellent detection tool for measuring the concentration of unreacted Au^3+^ at the ppt level. The concentration of the EW-AuNPs solution was measured by ICP-MS as 95,192.2 parts per billion (ppb) which was the initial Au^3+^ concentration used for the synthesis. The concentrations of the unreacted Au^3+^ were measured by ICP-MS as 8,455.6 and 7,151.1 ppb with the ultracentrifugation and filtration methods, respectively. Thus, the ultracentrifugation method obtained a yield of 91.1%, and the filtration method obtained a yield of 92.5%. The characteristic wine-red color of the EW-AuNPs disappeared after ultracentrifugation or filtration, indicating that the AuNPs were successfully separated from the unreacted Au^3+^.

### XRD analysis

The crystalline nature of the EW-AuNPs was determined via XRD analysis, as shown in Figure 1B. The diffraction peaks at 38.3°, 44.7°, 64.7°, and 77.4° corresponded to the (111), (200), (220), and (311) planes of crystalline Au, respectively, indicating a face-centered cubic structure.

### FT-IR spectra

As shown in Figure 1C, in the earthworm sample, the O-H stretching vibration appeared at 3,414 cm^−1^ as an intense and broad band. The two bands at 2,919 and 2,850 cm^−1^ were identified as the methylene vibrations of the hydrocarbons from the proteins/peptides [18]. The carbonyl (C = O) stretching vibration at 1,658 cm^−1^ from the amide functional groups also indicated the presence of proteins/peptides [18,19]. The band at 1,587 cm^−1^ resulted from the N-H bending vibration of the amide functional groups. The COO^–^ stretching vibration appeared at 1,412 cm^−1^. The bands from the earthworm sample suggested that proteins/peptides were the major compounds present in the sample. After synthesis of the EW-AuNPs, these bands shifted from 3,414 to 3,440 cm^−1^, from 2,919 to 2,914 cm^−1^, from 2,850 to 2,854 cm^−1^, from 1,658 to 1,637 cm^−1^, and from 1,412 to 1,406 cm^−1^. Based on these shifts, the proteins/peptides in the extract are likely responsible for the reduction of Au^3+^ to generate the AuNPs.

### HR-TEM images and size histogram

**Figure 2:**
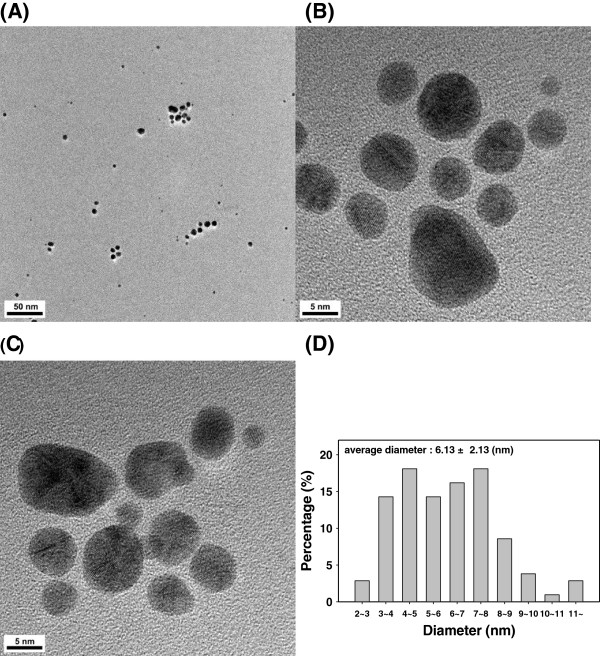
**HR-TEM images of the EW-AuNPs.** The scale bar represents **(A)** 50 nm, **(B)** 5 nm, and **(C)** 5 nm. **(D)** Size histogram.

The EW-AuNPs were primarily spherical with an average diameter of 6.13 ± 2.13 nm (Figure 2). A collection of 105 discrete AuNPs were randomly selected from the HR-TEM images to measure the average diameter. The two most abundant diameters were 4 ~ 5 and 7 ~ 8 nm, which accounted for 19% of the total (Figure 2D). Clear lattice fringes further confirmed the crystalline structure of the EW-AuNPs (Figure 2B,C). We previously obtained spherical EW-AuNPs with the diameter of 6.70 ± 2.69 nm using a green synthesis route with different reaction conditions [16].

### Anticoagulant activity via aPTT assay

**Figure 3:**
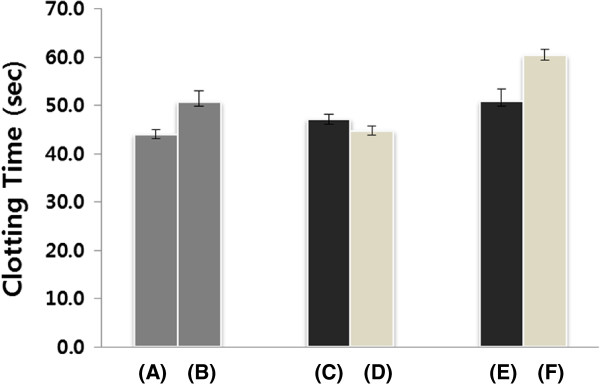
**Anticoagulant activity according to the aPTT assay.** The values in parentheses indicate the final concentrations of each component in the assay. **(A)** Negative control (deionized water), **(B)** positive control (heparin, 0.02 U/mL), **(C)** the extract (0.03%), **(D)** the EW-AuNPs (0.03% EW and 60 μM HAuCl_4_ · 3H_2_O), **(E)** a combination of heparin (0.02 U/mL) with sample **(C)**, and **(F)** a combination of heparin (0.02 U/mL) with sample **(D)**.

The EW-AuNPs reinforced or enhanced the anticoagulant activity of heparin by aPTT assay when the combination of EW-AuNPs and heparin was used for treatment (Figure 3). The clotting times of the negative (deionized water) and positive (heparin) controls were 44.1 and 50.8 s, respectively (Figure 3 parts A and B). No significant anticoagulant activities were noted in the extract (47.2 s, Figure 3 part C), the EW-AuNPs (44.8 s, Figure 3 part D), or in heparin combined with the extract (50.9 s, Figure 3 part E). However, when heparin and the EW-AuNPs were combined, the clotting time was extended to 60.4 s (Figure 3 part F), which corresponds to an increase of 118.9% and 134.8% over the clotting times of the same concentrations of the positive control (heparin) and the EW-AuNPs, respectively.

### AFM images

**Figure 4:**
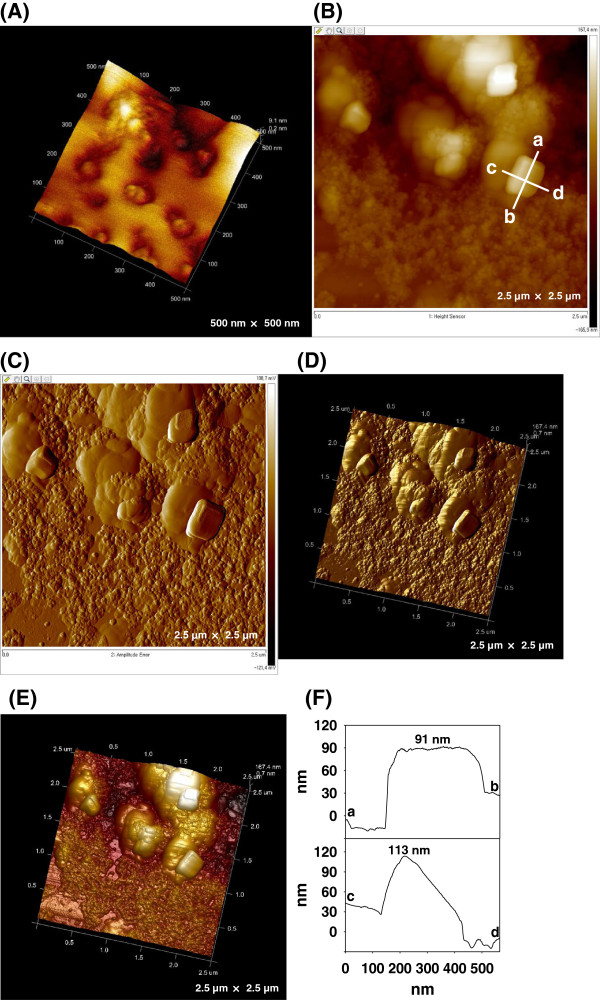
**AFM images of the EW-AuNPs. (A)** 3-D height image (500 nm × 500 nm), **(B)** 2-D height image (2.5 μm × 2.5 μm), **(C)** 2-D amplitude error image (2.5 μm × 2.5 μm), **(D)** 3-D amplitude error image (2.5 μm × 2.5 μm), **(E)** 3-D height image (2.5 μm × 2.5 μm), and **(F)** cross-sectional analysis of both the length (line a-b) and the width (line c-d) from B.

As depicted in Figure 4A, the obtained AuNPs were primarily spherical. This result is consistent with the HR-TEM images presented in Figure 2. Following an ultracentrifugation/resuspension process, the pellets (EW-AuNPs) were redispersed in deionized water and examined via AFM. The 2-D and 3-D images demonstrated that cubic and block-shaped AuNPs were also present as minor components (Figure 4B,C,D,E). Cross-sectional analysis further confirmed the block shape of the AuNPs (Figure 4F).

### FE-SEM images

**Figure 5:**
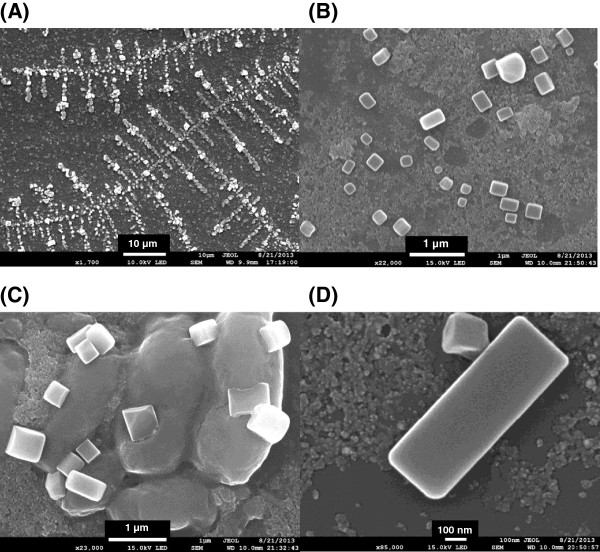
**FE-SEM images of the EW-AuNPs.** The scale bar represents **(A)** 10 μm, **(B)** 1 μm, **(C)** 1 μm, and **(D)** 100 nm.

When we imaged the cubic and block-shaped AuNPs via FE-SEM, these shapes appeared in a line that resembled fish bones (Figure 5A). A more detailed examination revealed cubic and block-shaped anisotropic particles. The width varied from 0.11 to 0.33 μm, and the length varied from 0.13 to 0.93 μm (Figure 5B). The aspect ratio is defined as the length divided by the width. The average aspect ratio was 1.47, with values ranging between 1.0 and 2.8. The largest observed block had a width of 0.33 μm, a length of 0.93 μm, and a maximum aspect ratio of 2.8 (Figure 5D). Murphy and co-workers reported that surfactants such as cetyltrimethylammonium bromide or small ions act as structure-directing agents in the formation of anisotropic nanostructures [20]. We hypothesize that the structure-directing agents in the extracts likely induced the formation of anisotropic shapes during synthesis. We previously reported the presence of glycosaminoglycans in these earthworm extracts [15]. Glycosaminoglycans are water-soluble compounds with large negative charges that can act as structure-directing agents. Based on the interpretation of the FT-IR spectra, proteins/peptides are the likely other candidates.

## Conclusions

We report the green synthesis of AuNPs using aqueous earthworm extracts as reducing agents to convert Au^3+^ to AuNPs and the characterization of these AuNPs. The reactions occurred in water without the use of any other toxic chemicals; thus, the resulting AuNPs were available for subsequent biological tests. Anisotropic NPs were observed in addition to the spherical NPs. We are unable to explain how the anisotropic NPs were generated, and this topic will be explored in future work. From the FT-IR spectra, we could conclude that the proteins/peptides in the extract were involved in the reduction of Au^3+^ and in the stabilization of the EW-AuNPs. In addition, the anticoagulant activity of heparin was reinforced when combined with the EW-AuNPs, which suggests that AuNPs are involved in the blood coagulation cascade. The current study demonstrates that the newly prepared AuNPs are promising candidates for novel gold nanomedicines.

## Competing interests

The authors declare that they have no competing interests.

## Authors’ contributions

HKK and SHJ performed the green synthesis of the EW-AuNPs. MJC, SHC, and YP characterized the EW-AuNPs. YKK performed the aPTT assay. SC and YP supervised the entire process and drafted the manuscript. All authors read and approved the final manuscript.
